# Effect of a Berry Polyphenolic Fraction on Biofilm Formation, Adherence Properties and Gene Expression of *Streptococcus mutans* and Its Biocompatibility with Oral Epithelial Cells

**DOI:** 10.3390/antibiotics10010046

**Published:** 2021-01-05

**Authors:** Mariem Souissi, Amel Ben Lagha, Kamel Chaieb, Daniel Grenier

**Affiliations:** 1 Laboratory of Analysis, Treatment and Valorization of Pollutants of the Environment and Products, Faculty of Pharmacy, University of Monastir, Monastir 5000, Tunisia; rayhanabio@hotmail.com (M.S.); chaieb_mo@yahoo.fr (K.C.); 2 Faculty of Sciences of Bizerta, University of Carthage, Zarzouna 7021, Tunisia; 3 Oral Ecology Research Group, Faculty of Dentistry, Université Laval, Quebec City, QC G1V 0A6, Canada; amelbenlagha@gmail.com

**Keywords:** anti-biofilm, anti-adhesion, berry polyphenols, epithelial cells, quorum sensing, *S. mutans*

## Abstract

The ability of *Streptococcus mutans* to adhere to oral surfaces and form biofilm is a key step in the tooth decay process. The aim of this study was to investigate a berry (wild blueberry, cranberry, and strawberry) polyphenolic fraction, commercialized as Orophenol^®^, for its antibacterial, anti-biofilm, and anti-adhesion properties on *S. mutans*. Moreover, the biocompatibility of the fraction with human oral epithelial cells was assessed. Phenolic acids, flavonoids (flavonols, anthocyanins, flavan-3-ols), and procyanidins made up 10.71%, 19.76%, and 5.29% of the berry polyphenolic fraction, respectively, as determined by chromatography and mass spectrometry. The berry polyphenolic preparation dose-dependently inhibited *S. mutans* biofilm formation while not reducing bacterial growth. At concentrations ranging from 250 to 1000 µg/mL, the fraction inhibited the adhesion of *S. mutans* to both saliva-coated hydroxyapatite and saliva-coated nickel–chrome alloy. Quantitative reverse transcription polymerase chain reaction (qRT-PCR) analysis showed that incubating *S. mutans* with the berry polyphenolic fraction was associated with a reduced expression of *luxS* gene, which regulates quorum sensing in *S. mutans*. The berry fraction did not show any significant cytotoxicity in an oral epithelial cell model. In conclusion, Orophenol^®^, which is a mixture of polyphenols from wild blueberry, cranberry and strawberry, possesses interesting anti-caries properties while being compatible with oral epithelial cells.

## 1. Introduction

Dental caries, the most common infection affecting the oral cavity and one of the most prevalent infectious diseases worldwide, is characterized by the enamel demineralization of the tooth [1,2]. Although a number of bacterial species of the dental plaque community have been associated with the cariogenic process, *Streptococcus mutans* is considered the principal etiological agent [2,3,4]. This Gram-positive facultative anaerobic bacterium is acid-tolerant and metabolizes dietary carbohydrates to produce high amounts of acids, including lactic acid, which has a strong demineralizing action [3]. The initial adhesion of *S. mutans* to dental surfaces is a prerequisite for biofilm formation, which also involves the synthesis of an extracellular polysaccharide matrix, known as glycans, by glycosyltransferases [3,5,6]. Biofilm formation allows *S. mutans* to be firmly attached and to trap acids close to the tooth surface, thus promoting the development of carious lesions.

Regular and appropriate oral hygiene procedures reduce the cariogenic biofilm and consequently decrease the severity and frequency of dental caries. In recent decades, research has been directed at identifying non-toxic edible products that can exhibit anti-caries properties. Polyphenols are compounds with a strong antioxidant activity and for which numerous human health benefits have been associated [7]. These plant secondary metabolites have been reported to possess antibacterial, anti-adhesion, and anti-inflammatory properties that may be of interest with regard to oral health [8,9]. Wild blueberry (*Vaccinium angustifolium*), cranberry (*Vaccinium macrocarpon*), and strawberry (*Fragaria virginiana)* are particularly rich in phenolic acids, proanthocyanidins and anthocyanins [10]. Previous studies have clearly shown that proanthocyanidins from berry fruits possess anti-biofilm and anti-adhesion properties against several oral pathogens [9,11,12]. The aim of this study was to investigate a commercial berry polyphenolic preparation (Orophenol^®^) made of wild blueberry, cranberry, and strawberry for its antibacterial, anti-biofilm, and anti-adhesion properties on the cariogenic bacterium *S. mutans*. Moreover, the biocompatibility of the berry fraction with human oral epithelial cells was assessed.

## 2. Results

The phenolic composition of the berry fraction, as determined by liquid chromatography–mass spectrometry, is reported in Table 1. Phenolic acids, flavonoids (flavonols, anthocyanins, flavan-3-ols), and procyanidins made up 10.71%, 19.76%, and 5.29% of the berry polyphenolic preparation, respectively. *p*-coumaric acid (42.19%) was the most abundant phenolic acid, while flavonols accounted for 95% of the total flavonoids, and quercetin and its sugar-conjugated derivatives for 83.87% of the flavonols. The procyanidin content was mostly monomers, dimers, and polymers with a degree of polymerization > 10.

**Figure 1:**
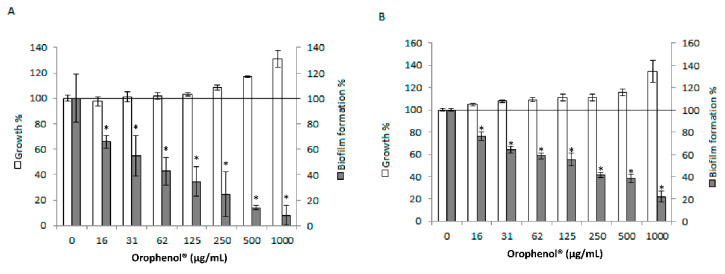
Effect of the berry polyphenolic fraction (Orophenol^®^) on growth and biofilm formation by *S. mutans* ATCC 25175 (Panel (**A**)) and *S. mutans* ATCC 35668 (Panel (**B**)). Results are expressed as the means ± SD of triplicate assays. *, significant decrease compared to the control assay in the absence of the berry fraction (*p* < 0.01).

The broth microdilution assay showed that the berry polyphenolic fraction, even at the highest tested concentration (1000 µg/mL), did not reduce the growth of the two strains of *S. mutans* (Figure 1). However, the fraction dose-dependently reduced the biofilm formation by *S. mutans* (Figure 1). More specifically, the minimal biofilm inhibitory concentration (MBIC) that inhibited the biofilm formation by at least 50% (MBIC_50_) and 90% (MBIC_90_) for *S. mutans* American Type Culture Collection (ATCC) 25175 were 62 and 500 µg/mL, respectively. For *S. mutans* ATCC 35668, the MBIC_50_ was 250 µg/mL while MBIC_90_ was > 1000 µg/mL. The inhibition of biofilm formation was not associated with a reduction in bacterial growth rate caused by the presence of the berry fraction. Indeed, monitoring the growth, in the presence and absence of the berry fraction, after 2, 6, 12, 18, 24 and 48 h of incubation did not show any reduction in bacterial growth (see Supplementary File).

**Figure 2:**
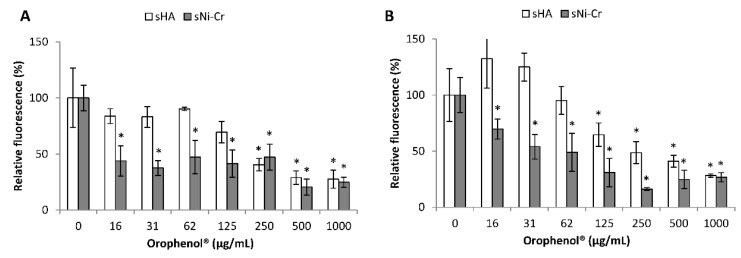
Effect of the berry polyphenolic fraction (Orophenol^®^) on the adherence of *S. mutans* ATCC 25175 (Panel (**A**)) and *S. mutans* ATCC 35668 (Panel (**B**)) to saliva-coated hydroxyapatite (sHA) and saliva-coated nickel–chrome alloy (sNi–Cr). Results are expressed as the means ± SD of triplicate assays. *, significant decrease compared to the control assay in the absence of the berry fraction (*p* < 0.01).

We then evaluated the ability of the berry polyphenolic fraction to inhibit the adherence of fluorescein isothyocyanate (FITC)-labeled *S. mutans* to saliva-coated hydroxyapatite (sHA) and saliva-coated nickel–chrome (sNi–Cr) alloy that mimicked dental enamel and cast restorations, respectively. As reported in Figure 2, the fraction more efficiently inhibited the adherence of *S. mutans* to sNi–Cr than to sHA. With regard to *S. mutans* ATCC 25175, the berry fraction at a concentration of 16 µg/mL reduced the adhesion to sNi–Cr by 56.2% while a concentration of 250 µg/mL was required to observe a comparable inhibition of the adherence to sHA. For *S. mutans* ATCC 35668, to obtain an inhibition ≥50%, concentrations of 62 and 250 µg/mL of the berry fraction were required for adhesion to sNI–Cr and sHA, respectively.

**Figure 3:**
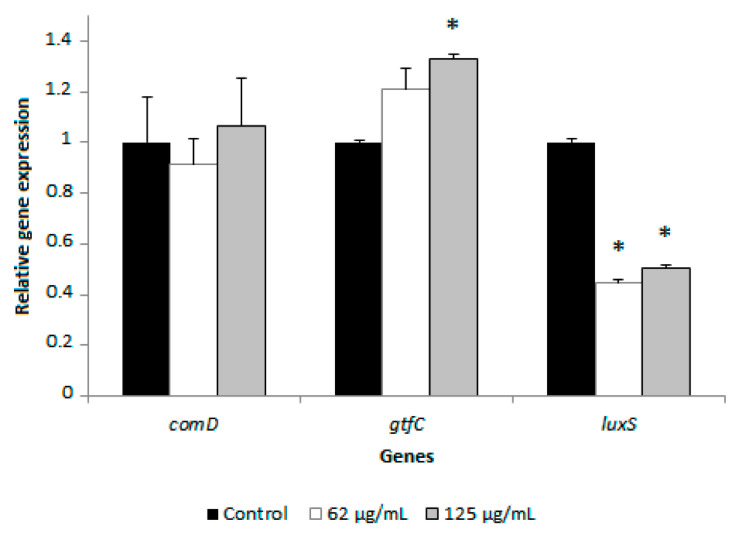
Effect of the berry polyphenolic fraction (Orophenol^®^) on the mRNA expression of *comD*, *gtfC*, and *luxS* in *S. mutans* ATCC 25175. The expression was normalized to 16S rRNA. Results are expressed as the means ± SD of triplicate assays. *, significantly different from the control assay in the absence of the berry fraction (*p* < 0.01).

We then investigated the effect of the berry polyphenolic fraction on the expression of three genes related to *S. mutans* biofilm formation, namely *luxS* and *comD* involved in quorum sensing, and *gtfC* implicated in glycan synthesis. For this purpose, *S. mutans* ATCC 25175 was incubated (4 h) with the fraction at concentrations of 62 and 125 µg/mL prior to quantify gene expression by quantitative reverse transcription polymerase chain reaction (qRT-PCR). As reported in Figure 3, in the presence of the berry fraction at 125 µg/mL, a significant decrease (50%) in the expression of *luxS* (autoinducer 2 synthase) was observed while the expression of *comD* (histidine kinase sensor protein) was not affected. A similar treatment of *S. mutans* increased *gtfC* (glycosyltransferase C) expression by 1.33-fold.

**Figure 4:**
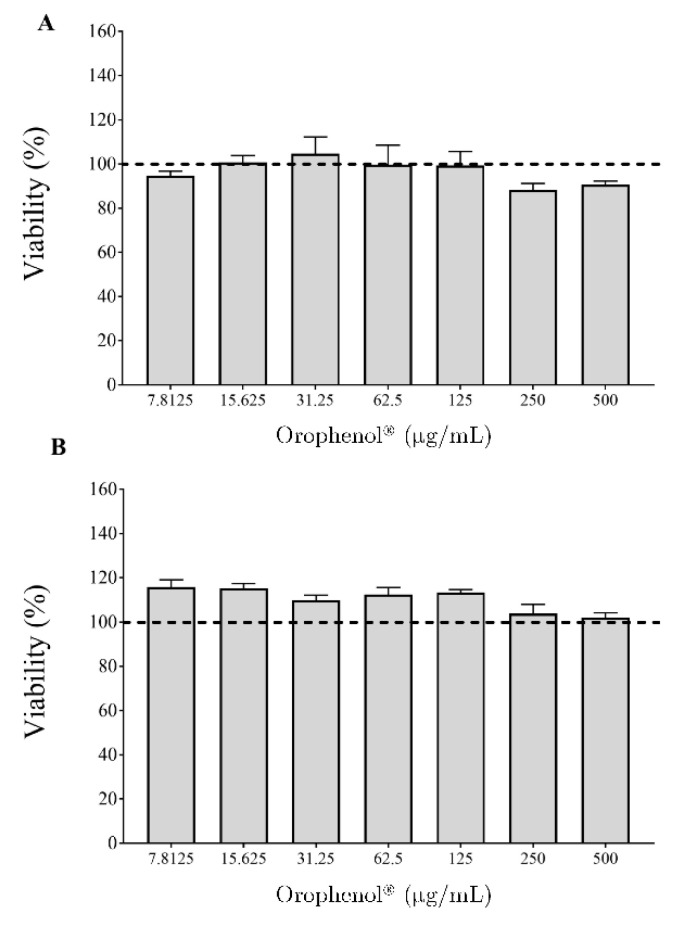
Effect of the berry polyphenolic fraction (Orophenol^®^) on the viability of human oral epithelial cells (B11 cell line) following 2 h (Panel (**A**)) and 48 h (Panel (**B**)) treatments. Results are expressed as the means ± SD of triplicate assays.

Lastly, the biocompatibility of the berry polyphenolic fraction was investigated using human oral epithelial cells (B11 cell line). Figure 4 shows that treating the epithelial cells during 2 and 48 h and the fraction did not significantly decrease the cell viability.

## 3. Discussion

Oral infections such as dental caries and periodontal diseases (gingivitis, periodontitis) are major public health problems because of their high prevalence and incidence in all regions of the world. As for other diseases, they mainly affect low economic status populations and minority groups, including aboriginal peoples, the elderly, and people who are cognitively and/or physically disabled. In industrialized countries, dental caries are the most common chronic disease of childhood, affecting 60–90% of school-aged children [1]. *S. mutans,* through its ability to adhere to the enamel surface, form biofilm, and produce demineralizing acids, is currently recognized as the key etiologic agent of tooth decay [2,3,4].

In recent years, a number of research groups brought evidence that plant polyphenols endowed with a capacity to act on different targets (pathogenic bacteria, host inflammatory response) may represent potential new therapeutic agents for the prevention/treatment of oral diseases [8,9]. More specifically, berry fruit proanthocyanidins have shown high potential for inhibiting bacterial adherence and biofilm formation [9,11,12,13]. In this study, we investigated the antibacterial, anti-biofilm, and anti-adhesion properties of Orophenol^®^, a commercial berry (blueberry, cranberry, and strawberry) polyphenolic fraction, on the cariogenic bacterium *S. mutans*.

Using a broth microdilution assay, the berry fraction did not show antibacterial activity against *S. mutans* at concentrations up to 1000 µg/mL. While we did not find previous studies investigating the antibacterial effect of blueberry and strawberry extracts on *S. mutans*, Duarte et al. [14] showed that cranberry polyphenols did not attenuate the growth of *S. mutans*.

The adhesion of *S. mutans* to dental surfaces is the initial step for biofilm formation by this cariogenic bacterium. The discovery of natural compounds with the ability to prevent the initial attachment of *S. mutans* to the tooth surface or dental prosthesis alloy is then of high interest to develop effective preventive therapy for dental caries. In this study, we showed that the berry polyphenolic fraction caused a significant inhibition of bacterial adherence to the sHA and sNi–Cr alloys that mimick dental enamel and cast restorations, respectively. It can be hypothesized that components of the berry fraction may modify the cell surface charge characteristics of *S. mutans* or hinder salivary receptors found on the HA or Ni–Cr surface. This is supported by the fact that plant polyphenols have been reported to interact with salivary proteins [15] as well as with the bacterial membranes that result in the modification of the hydrophobicity and charge of the cell surface [16]. While the adherence of *S. mutans* to the sNi–Cr alloy was significantly inhibited by the berry polyphenolic fraction at concentrations in the range of 16–125 µg/mL, no such inhibition was observed for adherence to sHA at those concentrations. This may be related to the fact that saliva has a much higher affinity for HA than for Ni–Cr. Consequently, higher numbers of bacteria can attach to sHA and therefore higher amounts of the berry polyphenolic fraction are required to cause a significant inhibition of adherence.

We also demonstrated that the berry fraction had a strong anti-biofilm activity on *S. mutans*. The dose-dependent decrease in biofilm formation was not associated with a reduction in bacterial growth rate caused by the presence of the berry fraction. This is of interest since the objective of anti-adhesion therapies in fighting dental caries is to inhibit the biofilm without affecting bacterial viability, thus minimizing the emergence of resistant strains. Studies on blueberry and cranberry polyphenols have shown their potential to inhibit the biofilm formation in a large array of Gram-positive and Gram-negative oral pathogens [13,17,18].

Quorum-sensing is a mechanism that allows bacteria embedded in biofilm to communicate through the secretion of small molecules [19]. This results in the modification of the expression of genes associated with virulence properties. In *S. mutans*, two signaling molecules have been identified, the competence-stimulating peptide (CSP) and the autoinducer-2 (AI-2) [20,21,22,23]. The *comD* gene encodes a histidine kinase sensor protein specific for the CSP that regulates the competence pathway in streptococci. The *luxS* gene encodes an enzyme (S-ribosyl-L-homocysteinase) involved in the production of 4,5-dihydroxy-2,3-pentanedione, the precursor of AI-2. qRT-PCR was then performed to investigate the effect of the berry polyphenolic fraction on the expression of genes related to biofilm formation in *S. mutans*. This analysis showed that the berry fraction significantly reduces the expression of *luxS* while having no effect on *comD* gene expression. This is in agreement with previous studies reporting that the *luxS* gene of *S. mutans* is involved in biofilm formation [24,25]. Interestingly, using a *luxS*-deficient mutant of *S. mutans*, Sztajer et al. [26] brought evidence that *luxS* is also involved in other functions, including carbohydrate metabolism, stress response, and acid tolerance. Although we observed that the berry fraction slightly increases the expression of the *S. mutans gtfC*, this has likely no impact on biofilm formation since the culture medium used in our biofilm assays did not contain sucrose, which is necessary for glycan formation by glycosyltransferases. Since the berry fraction does not reduce the expression of *gtfC*, it suggests that it does not target the formation of glycan.

Biocompatibility is a key parameter to consider when developing new compounds aimed to be used to prevent or fight oral infections. Interestingly, even at the highest tested concentration (500 µg/mL) and an exposure time of 48 h, the berry polyphenolic fraction did not reduce the cell viability of oral epithelial cells.

In conclusion, this study showed that a berry polyphenolic fraction, commercialized as Orophenol^®^ and made of wild blueberry, cranberry, and strawberry, exerts anti-adhesion and anti-biofilm properties on the cariogenic bacterium *S. mutans*. This further supports the potential of this preparation as an anti-caries agent since Philip et al. [27] recently reported that Orophenol^®^ reduces metabolic activity and lactic acid production in *S. mutans*. Given its biocompatibility with oral epithelial cells, our study suggests that the berry polyphenolic fraction offers potential in view of a novel alternative for preventing dental caries.

## 4. Materials and Methods

### 4.1. Berry Polyphenolic Fraction (Orophenol^®^) and Phenolic Characterization

The berry polyphenolic preparation, commercialized as Orophenol^®^, was kindly provided by Diana Food Canada Inc. (Champlain, QC, Canada). This fraction is made with polyphenol-rich soluble extracts prepared from wild blueberries, cranberries and strawberries. A stock solution (10 mg/mL) was prepared in distilled water, filtered through a 0.2 µm pore-size membrane filter and kept at 4 °C in the dark. Anthocyanins and procyanidins in the berry polyphenolic fraction were characterized by high-performance liquid chromatography (HPLC) as described previously [28]. Delphinidin 3-glucoside was used as a standard for anthocyanins. Procyanidins, which were eluted on the basis of their degree of polymerization, were quantified using epicatechin monomer as a standard. Phenolic acids and flavonoids were characterized as previously described [28] using an Acquity^®^ ultra-performance liquid chromatography–tandem mass spectrometer (UHPLC–MS/MS) coupled to a triple quadrupole (TQD) mass spectrometer equipped with a Z-spray electrospray interface (Waters Ltd., Mississauga, ON, Canada). External phenolic and flavonoid standards were analyzed using the same parameters and were used for the quantification.

### 4.2. Bacteria and Growth Conditions

Two strains of *S. mutans* (ATCC 25175 and ATCC 35668) were used in this study. Bacteria were grown at 37 °C in 1% tryptone + 0.5% yeast extract + 0.25% Na_2_HPO_4_ + 0.25% NaCl + 0.5% glucose (TYE-G).

### 4.3. Antibacterial Assay

Bacterial cells from overnight cultures of *S. mutans* were suspended in TYE-G to obtain a concentration corresponding to 1 × 10^8^ colony-forming units (CFU)/mL. Two-fold serial dilutions (2000–32 µg/mL) of the berry polyphenolic fraction in TYE-G (100 µL/well) were prepared in a 96-well tissue culture microplate and an equal volume of the bacterial suspensions was added. Control wells with no bacteria or no berry fraction were also prepared. Following a 24 h incubation at 37 °C, bacterial growth was monitored by recording the optical density at 660 nm (OD_660_) using a xMark microplate spectrophotometer (BioRad Laboratories, Mississauga, ON, Canada). Assays were performed in triplicate in three independent experiments and a representative set of data is presented.

### 4.4. Biofilm Formation Assay

The effect of the berry polyphenolic fraction on biofilm formation by *S. mutans* was investigated using the microplate assay with crystal violet staining as described by LeBel et al. [29]. Briefly, following bacterial growth (24 h) in a 96-well microplate, the spent medium and unattached bacteria were removed by aspiration and wells were washed twice with distilled water (100 µL) and then dried (37 °C) for 2 h. Biofilms were stained by adding 0.01% crystal violet (100 µL) to wells for 15 min. Wells were washed twice with distilled water to remove unbound crystal violet and the plates were dried at 37 °C. One hundred µL of 75% (*v*/*v*) ethanol was added to each well and the plates were shaken (15 min) to release the dye from the biofilms prior to monitoring the absorbance at 550 nm (A_550_) using a microplate reader. The minimal biofilm inhibitory concentration (MBIC) was defined as the lowest concentration of the berry fraction that inhibited biofilm formation by at least 50% (MBIC_50_) or 90% (MBIC_90_). Assays were performed in triplicate in three independent experiments and a representative set of data is presented.

### 4.5. Adherence Assays

#### 4.5.1. Saliva Collection

Parafilm-stimulated saliva was collected from six non-smoking healthy volunteers. Drinking and eating was not allowed during the 2 h period prior to saliva collection. Saliva samples (≈ 5 mL) were pooled, subjected to centrifugation (15,000× *g* for 10 min), filter-sterilized (0.45 µm pore-size membrane) and stored at −20 °C.

#### 4.5.2. Fluorescent Labeling of Bacteria

Bacteria from overnight cultures (10 mL) were harvested by centrifugation (7000× *g*) for 5 min and washed once in 50 mM phosphate-buffered saline (PBS; pH 7.2). Bacterial cells were suspended in 0.5 M sodium bicarbonate buffer pH 8.0 to an OD_660_ of 1.0, and 30 µL of fluorescein isothyocyanate (FITC) (10 mg/mL in 95% ethanol) were added to 10 mL of the bacterial suspensions to label cells with fluorescence as previously reported [30]. The bacterial suspensions were incubated at 37 °C in the dark with constant agitation during 60 min. FITC-labeled bacteria were harvested by centrifugation, washed three times in PBS, and suspended in the same buffer to obtain an OD_660_ of 0.5.

#### 4.5.3. Adherence to Saliva-Coated Hydroxyapatite

Wells of a 96-well microplate (black wall and black bottom; Greiner Bio One, Frickenhausen, Germany) were coated with hydroxylapatite (HA) according to the procedure described by Shahzad et al. [31]. Saliva (100 µL) was added to each HA-coated well and the plate was incubated for 30 min at room temperature prior to rinsing three times with PBS. FITC-labeled cells of *S. mutans* (100 µL; OD_660_ = 0.5) were added to the wells, along with the berry polyphenolic fraction at final concentrations ranging from 1000 to 16 µg/mL (in PBS). The plate was further incubated for 2 h at 37 °C under low agitation in the dark. Unbound bacteria were removed by aspiration, and the wells were washed twice with PBS. After adding 100 µL of PBS to each well, the relative fluorescence units (RUF; excitation wavelength 495 nm; emission wavelength 525 nm) corresponding to the level of bacterial adherence were determined using a Synergy 2 microplate reader (BioTek Instruments, Winooski, VT, USA). Control wells in the absence of the berry fraction were used to determine 100% adherence values while wells with no bacteria were used as controls to measure basal autofluorescence. Assays were performed in triplicate in three independent experiments and a representative set of data is presented.

#### 4.5.4. Adherence to Saliva-Coated Nickel–Chrome Alloy

Sterile squared pieces (1 cm × 1 cm; 2 mm thickness) of the nickel–chrome (Ni–Cr) alloy (Cr 13–16%/Si 2–4%/Cu 12–16%) were purchased from Shiva Products (Palghar, India). The Ni–Cr alloys were commercialized for making cast crown bridges as well as pontics and frames for hard resin crowns. Alloys were placed into wells of a 12-well microplate containing 200 µL/well of saliva. Plates were incubated with shaking (200 rpm) at 37 °C for 30 min prior to remove saliva by aspiration. FITC-labeled cells of *S. mutans* (500 µL/well; OD_660_ = 0.5) were added along with the berry polyphenolic fraction at concentrations ranging from 1000 to 16 µg/mL (in PBS). The plates were incubated in the dark at 37 °C during 1 h. The wells and Ni–Cr alloy samples were washed twice with PBS, prior to adding one mL of PBS and 200 µL of 1% Tween-80. After 5 min, aliquots of 100 µL were transferred into wells of a 96-well microplate (black wall and black bottom) and fluorescence was monitored as above. Control wells in the absence of the berry fraction were used to determine 100% adherence values while wells with no bacteria were used as controls to measure basal autofluorescence. Assays were performed in triplicate in three independent experiments and a representative set of data was presented.

### 4.6. qRT-PCR Analysis

To investigate the effect of the berry polyphenolic fraction on the expression of selected genes of *S. mutans* ATCC 25175 involved in biofilm formation, bacteria were grown to an OD_660_ = 0.1 and then the berry fraction (62.5 and 125 µg/mL) was added prior to further incubate at 37 °C for 4 h. Control cells were incubated in the absence of the berry fraction. Bacteria were collected by centrifugation (7000× *g* for 5 min) and treated with RNAprotect bacterial reagent (Qiagen Canada Inc., Montreal, QC, Canada). Bacterial samples were vortexed three times for 3 min and then washed with cold PBS. Bacteria were suspended in 10 mM Tris buffer containing EDTA (1 mM), lysozyme (15 mg/mL), mutanolysin (1000 U/mL) and proteinase K (20 mg/mL) and incubated at room temperature for 1 h. Sterile glass beads (diameter, 300–500 µm; Sigma-Aldrich Canada, Co., Oakville, ON, Canada) were then added and the bacterial suspensions were vortexed for 3 min. RNA was isolated and purified using the RNeasy minikit (Qiagen Canada Inc.). The amounts of mRNA were quantified with the Experion^TM^ system (Bio-Rad Laboratories). RNA from each sample (100 ng/µL) was reverse-transcribed using Maloney murine leukemia virus reverse transcriptase and random hexamers in a Bio-Rad MyCycler^TM^ thermal cycler (Bio-Rad Laboratories). Reverse transcription conditions were 5 min at 70 °C, 10 min at 25 °C, 50 min at 37 °C, and 15 min at 70 °C. Real-time PCR was used for the quantification of *comD*, *gtfC*, and *luxS* mRNA expression. The 16S rRNA gene was used as an internal control for data normalization. The primers used for the quantitative RT-PCR were purchased from Life Technologies Inc. (Burlington, ON, Canada) and are listed in Table 2. The sequences of primers were obtained from a previous study [30]. Reaction mixtures were prepared with 25 µL of PCR mixture containing 12.5 µL of IQ SYBR Green Supermix, 5 µL of cDNA, 1 µL of gene-specific primer, and 6.5 µL of RNase- and DNase-free water. The samples were amplified using a Bio-Rad MyCycler^TM^ thermal cycler (Bio-Rad Laboratories). The amplification conditions were 95 °C for 5 min followed by 30 cycles at 95 °C for 60 s, 58 °C for 60 s and 72 °C for 30 s. To validate the specificity of each primer pair, temperature curve analyses were performed. Assays were performed in triplicate in three independent experiments and a representative set of data is presented.

### 4.7. In Vitro Biocompatibility Assay with Oral Epithelial Cells

The human oral epithelial cell line B11, which has already been characterized [32] was kindly provided by S. Groeger (Justus Liebig University Giessen, Germany). Cells were cultured in keratinocyte serum-free medium (K-SFM; Life Technologies Inc.) supplemented with growth factors (50 µg/mL of bovine pituitary extract and 5 ng/mL of human epidermal growth factor) and 100 µg/mL of penicillin G–streptomycin. The cultures were incubated at 37 °C in a 5% CO_2_. To evaluate the effect of the berry polyphenolic fraction on cell viability, cells were seeded (1 × 10^5^ cells in 100 µL) into wells of a 96-well tissue culture plate and incubated at 37 °C in a 5% CO_2_ atmosphere until they reached confluence. Cells were treated with two-fold serial dilutions of the berry fraction (from 500 to 7.8 µg/mL) for 2 and 48 h. Thereafter, an MTT (3-[4, 5-diethylthiazol-2-yl]-2,5-diphenyltetrazolium bromide) assay was performed according to the manufacturer’s protocol (Roche Diagnostics, Mannheim, Germany) to assess cell viability. The assays were performed in triplicate and the means ± standard deviations were calculated.

### 4.8. Statistical Analysis

Statistical analyses were performed using the one-way ANOVA analysis of variance with a post hoc Tukey HSD test. The results were considered significant at *p* < 0.01.

## Figures and Tables

**Table 1:** Phenolic composition of the berry polyphenolic fraction. DP: degree of polymerization.

Composition	Amount (mg/100 g Dry Weight)
**PHENOLIC ACIDS**	**10,707**
Caffeic acid	611
Chlorogenic acid	1060
Cinnamic acid	551
*p*-coumaric acid	4517
Cryptochlorogenic acid	7
Ferulic acid	249
Gallic acid	117
*p*-hydroxybenzoic acid	948
Isoferulic acid	27
Neochlorogenic	64
Protocatechuic acid	1525
Salicylic acid	986
Sinapic acid	45
**FLAVONOIDS**	**19,756**
**Flavonols**	**18,769**
Isorhamnetin	902
Kaempferol	184
Kaempferol glucoside	693
Myricetin	145
Myricetin glucoside	486
Phlorizin	617
Quercetin	4917
Quercetin galactoside	3556
Quercetin glucoside	1627
Quercetin rhamnoside	3794
Quercetin rutinoside	1848
**Anthocyanins**	**450**
**Flavan-3-ols**	**537**
Catechin	393
Epicatechin	144
**PROCYANIDINS**	**5286**
Monomers	1530
Dimers	1721
Trimers	589
Tetramers	240
Pentamers	92
Hexamers	56
Heptamers	20
Polymers (DP > 10)	1038

**Table 2:** Primer sequences used for the real-time polymerase chain reaction analysis.

Genes	Primer Sequences		Product Size
***16S rRNA***	Sense	5′ CCATGTGTAGCGGTGAAATGC 3′	144
	Antisense	5′ TCATCGTTTACGGCGTGGAC 3′	
***comD***	Sense	5′ TTCCTGCAAACTCGATCATATAGG 3′	113
	Antisense	5′ TGCCAGTTCTGACTTGTTTAGGC 3′	
***gtfC***	Sense	5′ TTCCGTCCCTTATTGATGACATG 3′	122
	Antisense	5′ AATTGAAGCGGACTGGTTGCT 3′	
***luxS***	Sense	5′ CCAGGGACATCTTTCCATGAGAT 3′	147
	Antisense	5′ ACGGGATGATTGACTGTTCCC 3′	

## Data Availability

The data presented in this study are available on request from the corresponding author.

## Supplementary Materials

The following are available online at https://www.mdpi.com/2079-6382/10/1/46/s1, Table S1: Effect of the berry polyphenolic fraction on growth of *S. mutans* ATCC 25175, Table S2: Effect of the berry polyphenolic fraction on growth of *S. mutans* ATCC35668.
