# Supplementary material for: Effect of a Berry Polyphenolic Fraction on Biofilm Formation, Adherence Properties and Gene Expression of Streptococcus mutans and Its Biocompatibility with Oral Epithelial Cells
Source: Antibiotics (Basel). 2021 Jan 5;10(1):46. doi: 10.3390/antibiotics10010046 (PMC7824760; doi:10.3390/antibiotics10010046)
Supplement: Supplementary file 1 [file antibiotics-10-00046-s001.pdf]

Table S1. Effect of the berry polyphenolic fraction on growth of *S. mutans* ATCC 25175. Growth assays were performed in triplicate and a representative set of data is presented.

Berry fraction (µg/ml)	Growth (OD_{660})					
	2 h	6 h	12 h	18 h	24 h	48 h
0	0.02	0.16	0.72	1.03	1.01	0.92
62.5	0.02	0.18	0.75	1.02	1.02	0.88
125	0.02	0.19	0.73	1.00	1.0	0.95
250	0.01	0.15	0.70	1.10	1.12	1.10
500	0.02	0.14	0.78	1.15	1.15	1.09
1000	0.03	0.15	0.76	1.15	1.18	1.12

Table S2. Effect of the berry polyphenolic fraction on growth of *S. mutans* ATCC 35668. Growth assays were performed in triplicate and a representative set of data is presented.

Berry fraction (µg/ml)	Growth (OD_{660})					
	2 h	6 h	12 h	18 h	24 h	48 h
0	0.01	0.12	0.46	0.92	1.11	1.15
62.5	0.01	0.09	0.41	0.89	1.15	1.12
125	0.03	0.10	0.41	0.92	1.13	1.09
250	0.01	0.12	0.45	0.98	1.18	1.07
500	0.02	0.14	0.52	1.07	1.24	1.11
1000	0.03	0.13	0.52	1.12	1.28	1.18
